# Ordinary AV Nodal Reentry Tachycardia-Unusual Ablation: A Case Report and Review of the Literature

**DOI:** 10.1155/2022/9383016

**Published:** 2022-08-23

**Authors:** Franz Haertel, P. Christian Schulze, Anett Große, Dirk Prochnau, Ralf Surber

**Affiliations:** ^1^University Hospital Jena, Department of Internal Medicine I, Cardiology, Am Klinikum 1, 07743 Jena, Germany; ^2^Department of Internal Medicine, Cardiology, Sophien-und Hufeland-Klinikum, Henry-van-de-Velde-Straße 2, 99425 Weimar, Germany

## Abstract

A 72-year-old woman was referred to us with typical symptoms of paroxysmal supraventricular tachycardia for electrophysiological diagnostics and catheter ablation. During the first session of catheter ablation, a probing of the right ventricle was not successful. Therefore, an angiography of the central veins was performed. A rare anatomical variation with atresia of the inferior vena cava below the hepatic veins with azygos persistence was detected. The blood of the lower half of the body was drained via the dilated azygos into the superior vena cava; the blood of the liver veins enters into the right atrium directly. By atypical catheter placement over the azygos vein in the right ventricle and coronary sinus, an AV nodal reentry tachycardia (AVNRT) could be confirmed as the mechanism of tachycardia. However, a stable position of the ablation catheter could not be achieved by the femoral approach, so the successful AV node modulation with ablation of the slow pathway was performed via jugular access.

## 1. Introduction

Vena azygos persistence is considered an extremely rare, asymptomatic venous anomaly. As relevant anatomical access routes may not be available, particularly the transseptal access to the left atrium, it will pose a complex challenge for the interventionalist. With the number of cases of interventions gradually increasing, this anomaly must be given special consideration.

## 2. Case Report

**Figure 1:**
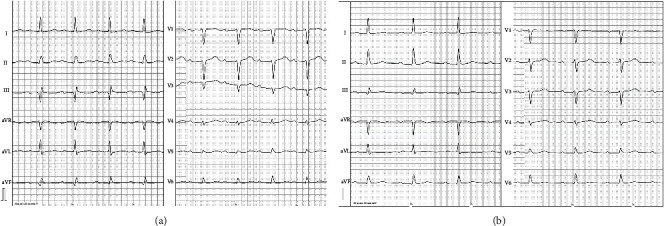
(a) 12-lead surface ECG before the intervention.(b) 12-lead surface ECG after the intervention with no change in the PQ interval.

A 72-year-old woman presented herself for a scheduled consultation in the outpatient clinic. During that meeting, both hypertensive derailment (blood pressure > 180/100 mmHg) and tachycardia with frequencies up to 200/min were noticed. A regular, supraventricular tachycardia (SVT) was diagnosed. The patient was referred to the central emergency department, where conversion to regular sinus rhythm was achieved by administration of adenosine. There was no ventricular preexcitation during sinus rhythm. Therefore, an atrioventricular nodal reentrant tachycardia (AVNRT) was suspected. The cardiac examination was uneventful with regular heart sounds with no pathological murmurs. Clinical examination of the respiratory tract, abdominal, and neurological status was unremarkable. Echocardiography revealed left ventricular hypertrophy with preserved systolic function, most likely due to hypertensive heart disease. No valvular disease was detected, and the dimensions of the atria were within normal range. In the current 12-lead electrocardiogram, we detected a normal sinus rhythm with a first-degree atrioventricular block (PR interval 216 ms) as shown in Figure 1.

**Figure 2:**
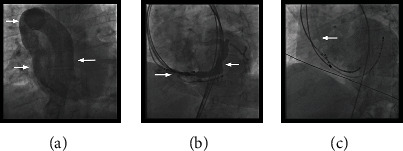
(a) Fluoroscopy of a dilated vena azygos (arrows) with inflow into the superior vena cava and the right atrium (p.a. projection). (b) Fluoroscopy of the coronary sinus (arrows), LAO 30°. (c) Fluoroscopy of the ablation catheter (arrow) via internal jugular vein placed in the area of the coronary sinus ostium (posteroseptal) during ablation, LAO 30°.

**Figure 3:**
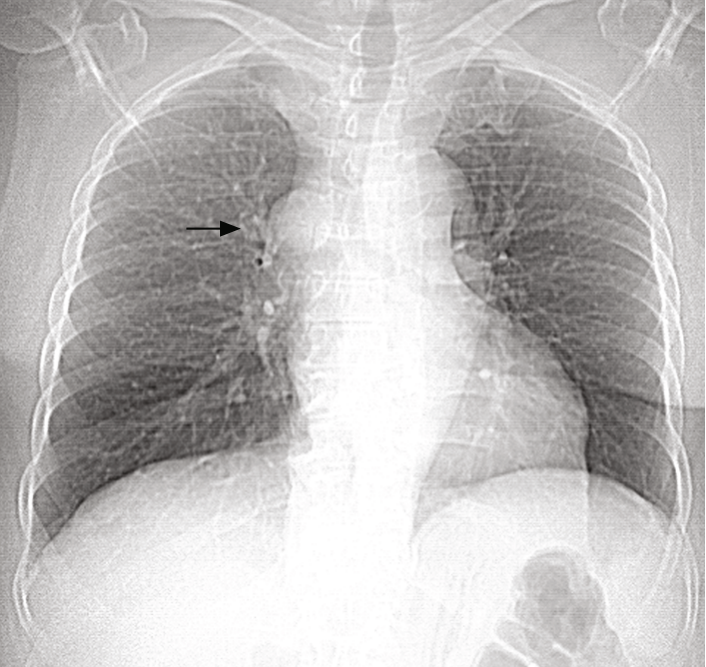
Chest radiograph showing a prominent upper right mediastinum (arrow) as the correlation of the azygos continuation entering the vena cava superior in a cross-sectional view.

**Figure 4:**
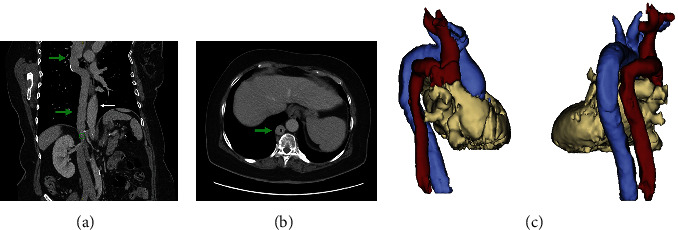
(a) Stretched reconstruction of a venous CT with azygos continuation (green arrows), aorta (white arrow), and diaphragmatic passage of the azygos (green circle). (b) Axial CT view, venous phase, showing a prominent azygos vein (green arrow). (c) 3D reconstruction showing the azygos continuation/superior vena cava (red) and the aortic arch (blue) in a right lateral and posterior view.

Subsequently, the patient was admitted to our cardiac unit for an elective ablation of the SVT. The patient gave informed consent to procedure and was brought to the electrophysiology laboratory. After establishing access to the femoral vein, an atypical anatomy of the large veins was suspected as the right atrium was not accessible conventionally. Next, a venous angiography was performed (Figure 2) which revealed that the coronary sinus was accessible through the azygos vein (Figure 2). Typical AVNRT was inducible as per standard protocol. However, a stable position of the ablation catheter could not be achieved by femoral access. As a consequence, the ablation could not be performed. A chest radiograph featured a suspicious configuration of the upper right mediastinum (Figure 3). Further diagnostics using contrast CT angiogram confirmed a rare anomaly: atresia of the inferior vena cava below the hepatic vein with azygos persistence (Figure 4).

**Figure 5:**
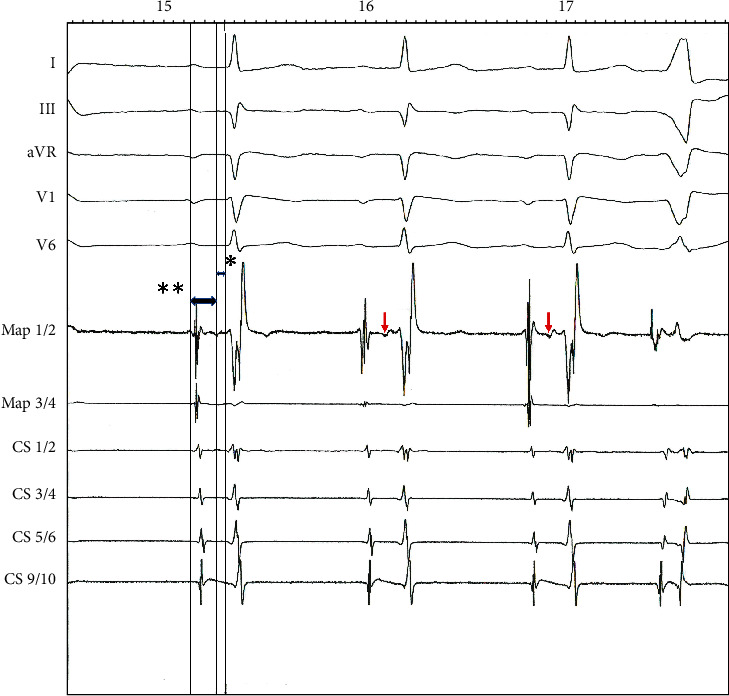
His bundle electrogram (red arrows) immediately performed before the second ablation attempt (^∗∗^atrioventricular (AV) interval of 128 ms; ^∗^His-ventricular (HV) interval of 48 ms).

**Figure 6:**
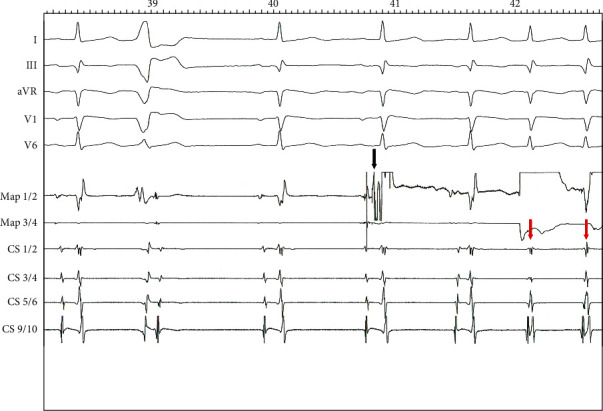
Electrogram of the ablation (black arrow: ablation start; red arrows: junctional beats after application of radiofrequency (RF)). A small A and a large V potential were recorded immediately before the ablation.

In a second session, catheter positioning in the coronary sinus was achieved via the right internal jugular vein and the catheter in the right ventricle via the femoral vein. The ablation catheter was also positioned via the internal jugular vein. Recording of His bundle activity prior to ablation could be achieved 15 mm above the ostium of the coronary sinus only with the ablation catheter and resulted in an atrioventricular (AV) interval of 128 ms and His-ventricular (HV) interval of 48 ms (Figure 5). A stable position of the ablation catheter was established in the same location posteroseptally at the ostium of the coronary sinus (Figure 2). The ablation was performed using a multicurve steerable ablation catheter (RF MARINR; Medtronic®) which enables variable curve size adjustment (range: from small tight curve to larger reach) and out-of-plane incremental lateral deflection including side-to-side tip rotation aiding placement in varied anatomy. This specific model used for the ablation is 7Fr and featured a 4 mm tip electrode with an embedded thermocouple temperature sensor. Tissue contact during ablation was adequate, verified by an impedance drop of >10% and occurrence of junctional beats during radiofrequency (RF) delivery in a typical right posteroseptal position at the ostium of the coronary sinus (Figure 6). Thereafter, no tachycardia could be induced, dual AV node physiology was no longer present, and the first-degree AV block remained unchanged (Figure 1). The patient was discharged without any complications; to this day, her clinical status remains stable with no recurrence of AVNRT.

## 3. Discussion

Vena azygos persistence usually occurs in combination with other congenital anomalies of the heart such as cyanotic or acyanotic congenital cardiac disorders and abnormalities of cardiac position such as dextrocardia and polysplenia or asplenia [1–3]. However, in this case, no other anomalies were found.

In the literature, a frequency ranging from 0.2 to 3.0% was reported as the incidence of systemic venous anomalies in patients undergoing cardiac catheterization [1, 4]. The most frequently seen variants are the persistence of the left superior vena cava, with or without a right superior vena cava, and azygos or hemiazygos continuation of an interrupted inferior vena cava [4]. For patients admitted to the cardiac unit of our hospital, an incidence of <0.2‰ was recorded for azygos continuation.

For symptomatic AVNRT patients, catheter ablation is currently considered as a standard treatment. Common access to the heart is attained from a femoral approach. The technique is proven to be safe and effective. However, individual cases of electrophysiologic examination and ablation of different arrhythmias have been described, demonstrating the feasibility of the ablation procedure under unusual anatomical circumstances, where even a transseptal approach with jugular access was attempted. As a particular challenge, the positioning and manipulation of the recording and mapping catheters are more difficult or even impossible due to their design, with a longer course, and angulation of the azygos vein connecting to the superior vena cava. At times, no electrical activity can be registered, and the catheter cannot be advanced to the His bundle position within the heart [5]. In the presented case, we could only achieve an effective position of the ablation catheter in a second ablation attempt from a combined jugular and femoral venous access that resulted in success.

Further cases of challenging ablation procedures in literature in which vascular access was not attainable using the standard approach by inserting the catheter into the femoral vein and advancing it through the inferior vena cava due to a prominent communication between the inferior vena cava and the superior vena cava via an azygos persistence include Wolff-Parkinson-White syndrome via right subclavian vein [5], atrioventricular junction ablation with an inferior approach for treatment of symptomatic, refractory atrial fibrillation in a VVIR-pacemaker patient [6], cryoablation for atrial fibrillation via right internal jugular vein using a transseptal sheath and BRK needle (Abbott Vascular) [7], ablation of AVNRT in a patient with dextrocardia after inserting the ablating catheter and two quadripolar catheters into the femoral and jugular veins [8], ablation of isthmus-dependent atrial flutter having the ablation catheter advanced from a femoral access [9], and ablation in a patient with coexistence of two different atrioventricular nodes (twin AV nodes) and AVNRT with venous access through the right internal jugular and left subclavian veins [10].

## 4. Conclusion

In the case of the above described anomaly, it was not possible to establish access to the right atrium via the femoral route. This anomaly should be put into consideration during procedures such as pulmonary vein isolation, left atrial appendage (LAA) occluder implantation, and interventional therapy on the mitral valve. The probing of the right atrium, ventricle, and coronary sinus is clearly more difficult from an inferior approach. In complex anatomical settings of electrophysiological cases, the use of a 3D mapping system should be considered.

## Data Availability

This is a case report. All data are included in the manuscript.

## Additional Points

*Learning Points*. With increasing percutaneous interventions, anatomical variations of the great veins should be considered. Probing and interventional procedures in the left atrium are not possible via the inferior vena cava in patients with azygos persistence. While they are possible through alternative routes via the internal jugular vein, they are challenging to perform and do not always result in success.
